# Glutamine promotes ovarian cancer cell proliferation through the mTOR/S6 pathway

**DOI:** 10.1530/ERC-15-0192

**Published:** 2015-08

**Authors:** Lingqin Yuan, Xiugui Sheng, Adam K Willson, Dario R Roque, Jessica E Stine, Hui Guo, Hannah M Jones, Chunxiao Zhou, Victoria L Bae-Jump

**Affiliations:** 1 Department of Gynecologic Oncology, ShanDong Tumor Hospital and Cancer Institute, Jinan University, Jinan, 250117, People's Republic of China; 2 Division of Gynecologic Oncology, University of North Carolina at Chapel Hill, CB #7572, Physicians Office Building Rm #B105, Chapel Hill, North Carolina, 27599, USA; 3 Lineberger Comprehensive Cancer Center, University of North Carolina at Chapel Hill, Chapel Hill, North Carolina, USA

**Keywords:** glutaminase, glutamate dehydrogenase, ovarian cancer, mTOR/S6, siRNA

## Abstract

Glutamine is one of the main nutrients used by tumor cells for biosynthesis. Therefore, targeted inhibition of glutamine metabolism may have anti-tumorigenic implications. In the present study, we aimed to evaluate the effects of glutamine on ovarian cancer cell growth. Three ovarian cancer cell lines, HEY, SKOV3, and IGROV-1, were assayed for glutamine dependence by analyzing cytotoxicity, cell cycle progression, apoptosis, cell stress, and glucose/glutamine metabolism. Our results revealed that administration of glutamine increased cell proliferation in all three ovarian cancer cell lines in a dose dependent manner. Depletion of glutamine induced reactive oxygen species and expression of endoplasmic reticulum stress proteins. In addition, glutamine increased the activity of glutaminase (GLS) and glutamate dehydrogenase (GDH) by modulating the mTOR/S6 and MAPK pathways. Inhibition of mTOR activity by rapamycin or blocking S6 expression by siRNA inhibited GDH and GLS activity, leading to a decrease in glutamine-induced cell proliferation. These studies suggest that targeting glutamine metabolism may be a promising therapeutic strategy in the treatment of ovarian cancer.

## Introduction

Ovarian cancer is the eighth most common cancer in women worldwide, with an estimated 21 980 new cases diagnosed and 14 270 deaths in the USA in 2014 (Siegel *et al*. 2014). Ovarian cancer is the most lethal of all the gynecologic malignancies in part due to the fact that patients are often diagnosed with advanced stage disease because of the absence of specific symptoms and the lack of adequate screening tests (Mirandola *et al*. 2011). There are no recognized preventative measures and no effective screening tools for ovarian cancer (Rooth 2013). The majority of primary ovarian malignancies are derived from epithelial cells, while ∼5% of ovarian carcinomas arise from other ovarian cell types (Sung *et al*. 2014). The standard of care for patients with advanced epithelial ovarian carcinoma involves an attempt at optimal cytoreductive surgery followed by chemotherapy with a platinum based regimen in combination with paclitaxel. However, although most ovarian tumors are very sensitive to this chemotherapy regimen, the large majority of patients develop a recurrence as well as drug resistance (Kajiyama *et al*. 2012, Rauh-Hain *et al*. 2013). Therefore, new cytotoxic treatment strategies are urgently needed to improve outcomes in patients with ovarian cancer.

Cancer cells have unique metabolism characteristics such as elevated energetic and biosynthetic demands of rapid cell growth and proliferation. The most well-known characteristic of cancer cell metabolism is the Warburg effect, in which cancer cells consume large amounts of glucose through the glycolysis pathway to produce ATP and lactate in the presence of oxygen (Bayley & Devilee 2012, Kee & Cheong 2014). Although cancer cells exhibit high rates of glycolysis, their mitochondrial oxidative phosphorylation (OXPHOS) remains intact and becomes progressively more dependent on glutamine metabolism (Hammoudi *et al*. 2011). Glucose and glutamine are the two major nutrient inputs for cancer cells as they provide bioenergetics and intermediates for macromolecular synthesis in proliferating cancer cells. The rate of conversion of glutamine to lactate is higher in cancer cells than in normal cells, which represents an alternative metabolic pathway to glucose consumption in a glucose-limiting environment (Helmlinger *et al*. 2002). The activity of glutaminase (GLS), the first enzyme in glutaminolysis, and glutamine level in the medium correlate with cancer cell proliferation. Serum glutamate levels are associated with tumor aggressiveness (Koochekpour *et al*. 2012). The function of glutamine to promote cell growth is likely dependent on their genetic and epigenetic background (Simpson *et al*. 2012, Phang *et al*. 2013). The restriction of glutamine induces apoptosis in melanoma and prostate cancer cells and the inhibition of glutaminolysis by acivicin has been shown to be very effective in the treatment of animal models of cancer (Fu *et al*. 2004, 2010, Roy & Maity 2005). In addition, a recent study showed that the inhibition of glutamine uptake is a promising new therapeutic strategy for treating acute myeloid leukemia (Willems *et al*. 2013). However, the impact of glutamine deprivation on ovarian cancer cell proliferation is less well characterized.

Given that glutamine metabolism is up-regulated in cancer cells, this study aimed to investigate the effects of glutamine on cell proliferation in ovarian cancer cells, and determine its underlying molecular mechanisms. We observed that depletion of glutamine inhibited cell growth, induced significant apoptosis, caused cell cycle G1 arrest, and induced reactive oxygen species (ROS) production in ovarian cancer cells. Moreover, glutamine increased cellular glycolytic activity and stimulated cell proliferation by modulating the mTOR/S6 pathway. These results indicate that targeting glutamine metabolism is a promising therapeutic strategy for ovarian cancer.

## Materials and methods

### Cell culture and reagents

The human ovarian cancer cell lines HEY, SKOV3, and IGROV-1 were used. The HEY cells were kindly provided by Dr McAsey of Department of Obstetrics and Gynecology at Southern Illinois University School of Medicine. The SKOV3 and IGROV-1 cell lines were provided by Dr Jazaeri from the Department of Obstetrics and Gynecology, University of Virginia. All three cell lines have been authenticated to be ovarian cancer cell lines (Yoshioka *et al*. 2012, Jazaeri *et al*. 2013, Xie *et al*. 2013, Ayyagari & Brard 2014). The cell lines used in this study were passaged for fewer than 6 months after resuscitation and the passage numbers were 5–20. The HEY and IGROV-1 cell lines were maintained in RPMI-1640 medium supplemented with 5 and 10% fetal bovine serum (FBS) respectively. The SKOV3 cell line was maintained in DMED/F12 medium supplemented with 10% FBS. For glutamine studies, the cells were cultured in RPMI-1640 medium or DMED/F12 medium without glutamine (cat #21870-076 and 12634-010, Gibco) containing 5% dialyzed FBS and supplied with various concentrations of glutamine. All media were supplemented with 100 U/ml of penicillin and 100 μg/ml of streptomycin. The cells were cultured in a humidified 5% CO_2_ at 37 °C.

l-glutamine was purchased from Corning Cellgro (Manassas, VA, USA). 3-(4,5-Dimethyl-2-thiazolyl)-2,5-diphenyl-2H-tetrazolium bromide (MTT), RNase A, bromopyrvic acid (3-BP), 2′,7′-dichlorofluorescin diacetate (DCFH-DA), and rapamycin were purchased from Sigma–Aldrich. The l-Lactate Assay Kit was bought from Eton Bioscience (San Diego, CA, USA). 2-[*N*-(7-nitrobenz-2-oxa-1,3-diazol-4-yl)amino]-2-deoxy-d-glucose (2-NBDG) and ATP assay kit were bought from AAT Bioquest (Sunnyvale, CA, USA). The glutamate dehydrogenase (GDH) assay kit was bought from BioVision (Milpitas, CA, USA). The RPS6 siRNA was purchased from Ambion Life Technologies. The HiperFect Transfection Reagent was bought from Qiagen. Annexin-V FITC Kit was purchased from Biolegend (San Diego, CA, USA). The anti-GLS antibody was purchased from Abcam (Cambridge, MA, USA), and all the other antibodies were obtained from Cell Signaling (Danvers, MA, USA). ECL detection reagents were purchased from GE Health care (Piscataway, NJ, USA). All other chemicals were purchased from Sigma.

### Cell proliferation assay

The ovarian cancer cells, HEY, SKOV3, and IGROV-1, were seeded at 3000 cells/well in 96-well plates in their culture media. After 24 h, cells were cultured in media with different concentrations of glutamine for 48 h. Cell proliferation was measured by adding 5 μl of MTT solution (5 mg/ml) per well for an additional incubation of 1 h. The MTT reaction was terminated through the replacement of the media by 100 μl of DMSO. Viable cell densities were determined by measuring absorbance of metabolic conversion of the colorimetric dye at 570 nm. Each experiment was performed in triplicate and repeated three times to assess for consistency of results.

### Cell cycle analysis

The effect of glutamine on cell cycle progression was assessed by using Cellometer (Wang *et al*. 2014; Nexcelom, Lawrence, MA, USA). Cells were plated at a density of 1.5×10^5^ cells/well in six-well plates overnight, and then treated with various concentrations of glutamine for 48 h. Cells were collected by 0.05% Trypsin (Gibco), washed with PBS solution, fixed in a 90% methanol solution and then stored at −20 °C until cell cycle analysis was performed. On the day of analysis, the cells were washed with PBS and centrifuged, resuspended in 50 μl RNase A solution (250 μg/ml) with 10 mM EDTA, followed by incubation for 30 min at 37 °C. After incubation, 50 μl of propidium iodide (PI) staining solution (2 mg/ml PI, 0.1 mg/ml azide, and 0.05% Triton X-100) was added to each tube and incubated for 10 min in the dark. The cell cycle was detected by Cellometer. The cell cycle progression was analyzed by the FCS4 Express Software (Molecular Devices, Sunnyvale, CA, USA).

### Annexin-V assay

The effect of glutamine on cell apoptosis was detected by using Annexin-V FITC Kit. Briefly, 1.7×10^5^ cells/well were seeded into six-well plates overnight, and then the cells were cultured in the media with various concentrations of glutamine for 24 h. The cells were collected by 0.25% Trypsin without EDTA. After PBS washing, the cells were resuspended in 100 μl of Annexin-V and PI dual-stain solution (0.1 μg of Annexin-V FITC and 1 μg of PI) for 15 min in the dark and detected by Cellometer. The results were analyzed by the FCS4 Express Software. Each experiment was repeated at least twice for consistency of response.

### Glucose uptake assay

Glucose uptake assay was performed using 2-NBDG. In brief, after cells were seeded in 96-well plates at 3000 cells/well in their culture media overnight, the cells were treated in various concentrations of glutamine (0, 0.5, 2.0, and 5.0 mM) for 24 h and then the cells were cultured with 2-NBDG (100 μg/ml) in glucose-free media with matching concentrations of glutamine for 40 min. The 2-NBDG uptake reaction was stopped by removing the media and washing the cells twice with 200 μl HBSS. Fluorescence (excitation/emission=485/535) was measured using a plate reader (Tecan, Morrisville, NC, USA). Data were normalized based on the viable cell counts measured by the MTT assays. All the experiments were performed in triplicate and repeated twice.

### l-lactate assay

l-lactate in the medium was detected by using the l-Lactate Assay Kit. Briefly, after we treated cells with different concentrations of glutamine for 24 h, 10 μl of the culture medium was transferred into a new 96-well plate and 40 μl of distilled water was added to each well. Another 50 μl of lactate assay solution was also then added to each well and the plates were incubated for 30 min at 37 °C without CO_2_. The lactate level was measured at wavelength of 490 nm using a plate reader from Tecan. The experiments were performed in triplicate and repeated twice to assure consistency.

### ATP assay

Cellular ATP production was assessed by using Luminometric ATP Assay Kit. In short, after we treated cells with glutamine for 24 h, 90 μl/well of the ATP reaction mix was added into the sample and mixed gently, and then incubated for 10–20 min in the dark at room temperature. The luminescence intensity was monitored with a plate reader from Tecan. The ATP levels were normalized based on the viable cell counts measured by the MTT assays. Each experiment was performed in triplicate and repeated twice to assess for consistency of results.

### ROS assay

Intracellular ROS production was detected using DCFH-DA (Wang *et al*. 2014). DCFH-DA can diffuse into cells and be deacetylated by cellular esterases to non-fluorescent DCFH, which cannot get through membranes freely and be rapidly oxidized to highly fluorescent DCFH by ROS. After we finished the treatment of the cells, 10 μl of 200 μM of DCFH-DA was added into the media and mixed gently. The fluorescence intensity was measured at an excitation wavelength of 485 nm and an emission wavelength of 530 nm using a plate reader from Tecan. Data were normalized based on the viable cell counts measured by the MTT assays. Each experiment was repeated at least twice for consistency of response.

### GDH activity assay

Intracellular GDH activity was measured by using the GDH assay kit. Cells were seeded into six-well plates at 1.5×10^5^ cells/well overnight, and then the cells were cultured in media with various concentrations of glutamine for 24 h. Cell lysates were prepared in cold GDH assay buffer. After 10–20 μl of cell lysates were transferred into a new 96-well plate, then the GDH assay buffer was added until the total volume was 50 μl. Composed of 82 μl of GDH assay buffer, 8 μl of GDH developer and 10 μl of glutameate, and 100 μl of reaction mix was added to each well. The concentration of GDH was measured at a wavelength of 450 nm in a plate reader after incubating for 3 min at 37 °C. The results were normalized on the basis of the total protein concentration of each sample. The experiments were performed in triplicate and repeated twice to assess for consistency of results.

### siRNA transfection

Transfection of siRNA was performed using Ambion RPS6 siRNA and Qiagen HiperFect Transfection Reagent. In brief, HEY cells were plated at a density of 1×10^5^ cells/well in 12-well plates or 5×10^3^ cells/well in 96-well plates. The transfection complexes containing 137.5 ng of RPS6 siRNA and 6 μl HiperFect Transfection Reagent for 12-well plate cells or 25 ng of RPS6 siRNA and 1 μl of HiperFect Transfection Reagent for 96-well plate cells were added into the media. A scramble siRNA was used as the negative control. The impact of siRNA transfection on cell proliferation was evaluated by MTT assay. The experiments were repeated twice.

### Western blot analysis

Total protein was extracted from ovarian cancer cells using RIPA buffer and the protein was quantified with the BCA Assay Kit (Thermo Scientific, Rockford, IL, USA). Protein samples with equal loading (30 μg/well) were separated by 10–12% SDS–PAGE and transferred onto PVDF membranes. The membranes were blocked with 5% nonfat milk and then incubated with a 1:1000 dilution of primary antibodies overnight at 4 °C. The membranes were washed and incubated with a secondary peroxidase-conjugeted antibody for 1 h at room temperature. The membranes were developed using an enhanced ECL at Alpha Innotech Imaging System (Protein Simple, Santa Clara, CA, USA). After developing, the membranes were re-probed using antibody against α-tubulin to confirm equal loading. Each experiment was repeated at least twice for consistency of results.

### Statistical analysis

Data is expressed as mean±s.e.m. Data was compared using two-tailed Student's *t*-test, and *P*<0.05 was considered significant.

## Results

### Glutamine promotes cell growth in ovarian cancer cells

**Figure 1:**
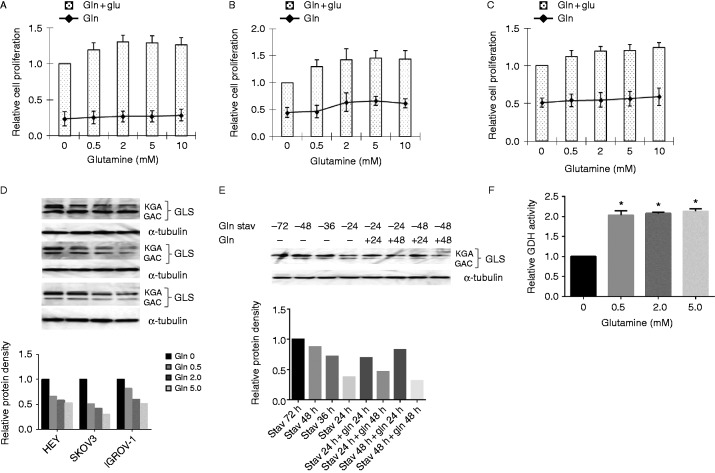
Glutamine metabolism promotes optimal cell proliferation. Ovarian cancer cells lines HEY (A), SKOV3 (B), and IGROV-1 (C) were treated in glutamine-free media supplemented with various concentrations of glutamine (0, 0.5, 2.0, 5.0, and 10.0 mM) for 48 h. Cell proliferation was assessed by MTT assay. The changes in glutaminase (GLS) expression upon the effects of glutamine were assessed using western blot. HEY, SKOV3, and IGROV-1 cells were treated with glutamine for 24 h. Treatment of glutamine reduced the expression of GLS in a dose-dependent manner (D). GLS changes in IGROV-1 cells after treatment with glutamine and glutamine starvation: glutamine starvation consistently increased the expression of GLS with the extension of starvation, and glutamine reduced the expression of GLS with the presence of glutamine for 48 h after starvation in IGROV-1 cells (E). GDH activity was assayed using a GDH assay kit in IGROV-1 cells. Glutamine greatly increased the activity of GDH (F). Data is shown as mean±s.e.m. of two trials (*P<0.05).

Glucose and glutamine are essential energy and nutrient sources for the growth and survival of cancer cells. To verify the effect of glutamine on the growth of ovarian cancer cells, three ovarian cancer cell lines, HEY, SKOV3, and IGROV-1, were treated in glutamine-free media with various concentrations of glutamine (0, 0.5, 2.0, 5.0, and 10.0 mM) for 48 h. Cell proliferation was assessed by MTT assay. The results showed that treatment of cells with glutamine in the media for 48 h increased cell proliferation by 20–50% in a dose-dependent manner. To further validate the energy source of cell growth in ovarian cancer cells, the three cell lines were treated with the same concentration of glutamine in the absence of glucose in the culture media. The results showed that increasing concentrations of glutamine slightly increased cell proliferation in 48 h in all three cell lines (Fig. 1A, B and C). Thus, these results confirm that glucose is a critical nutrient for ovarian cancer cells and that glutamine promotes optimal cell proliferation in glucose supplying conditions.

GLS catalyzes the hydrolysis of l-glutamine to l-glutamate, which is involved in oxidation in the mitochondria. We next investigated the impact of glutamine on GLS protein expression using different levels of glutamine in our ovarian cancer cells. The antibody recognizes two transcript variants of GLS1: KGA and GAC (Gross *et al*. 2014). Surprisingly, we found that treatment of cells with glutamine for 24 h reduced the expression of GLS in a dose-dependent manner (Fig. 1D). To further verify the effects of glutamine on GLS expression, IGROV-1 cells, a glutamine dependent cell line, were cultured under glutamine starvation for 24, 36, 48, and 72 h, and then treated cells with 2.0 mM glutamine in regular growth medium for 24 and 48 h. The results of western blot showed that glutamine starvation consistently increased the expression of GLS with the extension of starvation, and treatment of glutamine for 24 h increased the expression of GLS protein whereas glutamine reduced the expression of GLS after 48 h treatment at either 24 or 48 h starvation (Fig. 1E). Thus, these results indicate that the role of GLS in glutamine conversion is a rate-limiting process, and the activity of GLS controlled the flux of glutaminolysis (Qie *et al*. 2014). To help validate these results, we further assessed the activity of GDH in the presence or absence of glutamine. GDH converts glutamate to α-ketoglutarate in glutamine catabolism. We observed that there is a strong correlation between increased GDH activity and presence of glutamine after 24 h of treatment (Fig. 1F). These results suggest that glutamine promotes glutaminolysis, which supports cell growth in ovarian cancer cells.

### Glutamine regulates cell cycle and apoptosis in ovarian cancer cells

**Figure 2:**
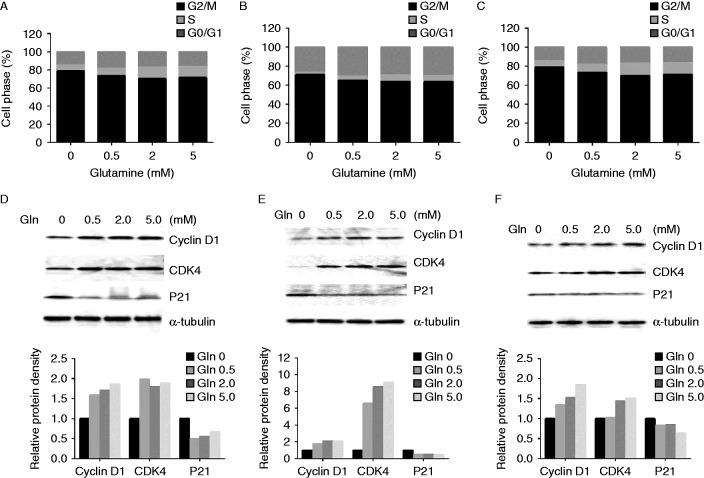
Glutamine affects cell cycle progression. HEY (A), SKOV3 (B), and IGROV-1 (C) cells were treated in glutamine-free media supplemented with various concentrations of glutamine (0, 0.5, 2.0, and 5.0 mM) for 48 h. Cell cycle analysis was performed using Cellometer. Depletion of glutamine induced cell cycle G1 phase arrest in ovarian cancer cells. The effects of glutamine on cyclin D1, CDK4, and p21 were examined by western blotting in HEY (D), SKOV3 (E), and IGROV-1 (F) cells after exposure to glutamine for 24 h at the indicated concentrations.

To elucidate the mechanisms of glutamine on cell growth, the effects of glutamine on cell cycle progression were analyzed. The three ovarian cancer cell lines were cultured with various concentrations of glutamine (0, 0.5, 2.0, and 5.0 mM) for 48 h, and the cell cycle changes were analyzed using Cellometer. The results showed that depletion of glutamine induced cell cycle G1 arrest in the three cell lines. The S phase gradually increased with increasing concentrations of glutamine in the three cell lines (Fig. 2A, B and C). These results indicated that glutamine promoted cell growth by inducing cell cycle changes. We further investigated the effects of glutamine on cell cycle checkpoints. Cyclin D1 activates CDK4 and promotes the passage of the cell into S phase from G1, and p21 is a key regulator for the cycle checkpoint of G1/S (Lenz *et al*. 2012). The results of western blot showed that the expressions of both cyclin D1 and CDK4 increased, and p21 decreased with increasing glutamine concentrations for 24 h (Fig. 2D, E and F). These results suggest that glutamine promotes the passage of cells into S phase from G1.

**Figure 3:**
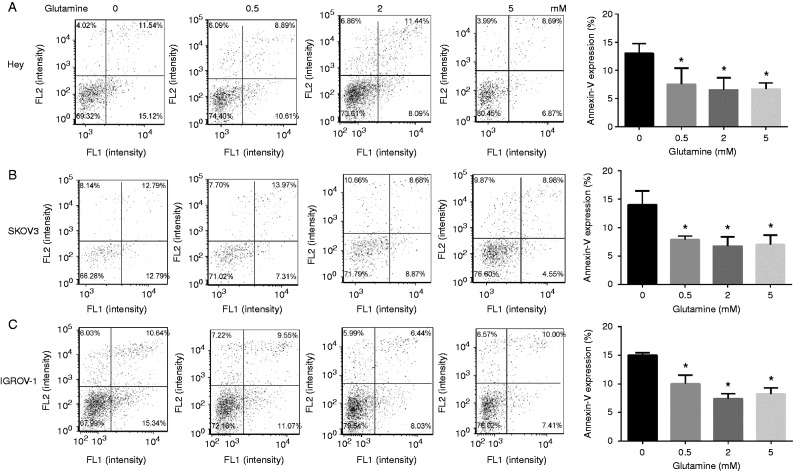
Depletion of glutamine induces cell apoptosis. HEY (A), SKOV3 (B), and IGROV-1 (C) cells were cultured with different concentrations of glutamine for 24 h. The apoptosis were detected using an Annexin-V FITC Kit. Depletion of glutamine induced significant cell apoptosis in the three cell lines. Data is shown as mean±s.e.m. of triplicates (*P<0.05).

Finally, we evaluated the effects of glutamine on cell apoptosis using Annexin-V assay. Annexin-V binds to phosphaticylserine externalized on the surface of the cell membrane, which is a distinct phenomenon of early apoptosis (Schointuch *et al*. 2014). The percentage of apoptotic cells increased distinctly in glutamine-free media in all three cell lines after 24 h of treatment (Fig. 3A, B and C), suggesting that depletion of glutamine in regular media could induce significant apoptosis in ovarian cancer cells.

### Glutamine deprivation induces cell stress

**Figure 4:**
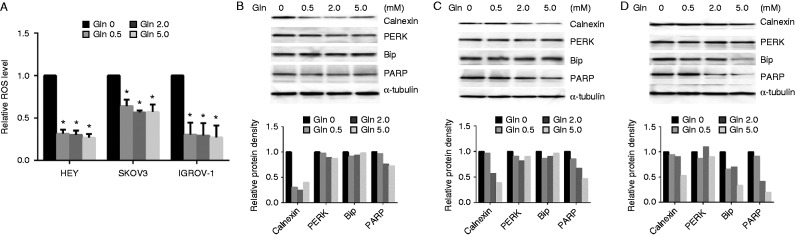
Depletion of glutamine induces cell stress. The three cell lines were treated with glutamine for 24 h. The production of intracellular reactive oxygen species (ROS) was detected using DCFH-DA. Depletion of glutamine greatly increased ROS production (A). The expression of stress proteins in cells was detected using western blotting. Glutamine reduced the expression of stress proteins in HEY (B), SKOV3 (C), and IGROV-1 (D). *P<0.05.

Increased ROS is an index of intracellular oxidative stress. To investigate whether different concentrations of glutamine induced cell stress, we determined the effects of glutamine on ROS production using DCFH-DA assay. The depletion of glutamine greatly increased ROS production by 1.5- to 3.6-fold in the three cell lines after 24 h. The addition of glutamine in the media markedly reduced ROS production (Fig. 4A). We then analyzed the changes in proteins related to cell stress, including protein kinase-like endoplasmic reticulum kinase (PERK), poly (ADP-ribose) polymerase (PARP), Calnexin, and Bip using western blotting after the cells were treated with different concentrations of glutamine for 24 h. The data showed that depletion of glutamine enhanced the expression of PERK, PARP, Bip, and Calnexin, but the expression of these proteins significantly decreased in the presence of glutamine (Fig. 4B, C and D). These results indicate that glutamine reduces intracellular oxidative stress and ER stress induced by depletion of glutamine.

### Glutamine complements ATP production on the basis of glycolysis

**Figure 5:**
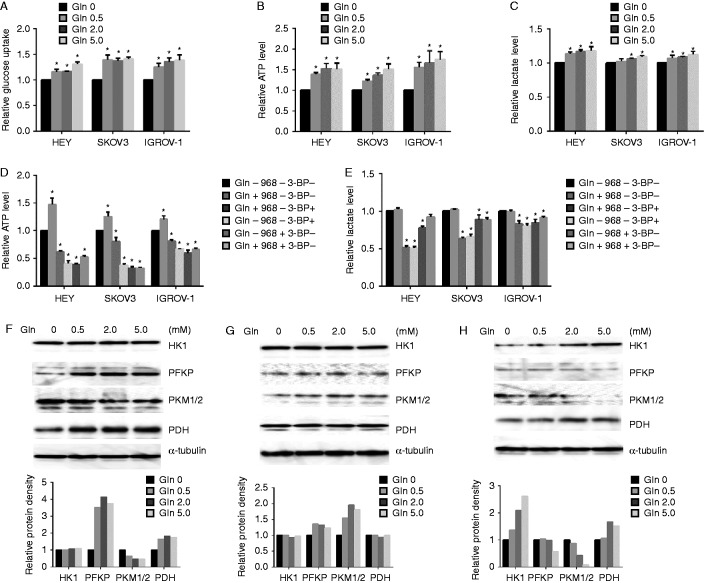
Glutamine complements ATP production. The cells were cultured in media with various concentrations of glutamine for 24 h. The levels of cellular glucose uptake (A), cellular ATP production (B), and lactate in the media (C) were detected. The cells were treated with compound 968 or 3-BP for 24 h in 2.0 mM glutamine or glutamine-free media respectively. The levels of ATP (D) and lactate (E) were detected by ELISA assay. The expression of glycolytic proteins was detected by western blotting in HEY (F), SKOV3 (G), and IGROV-1 (H) after treatment with glutamine for 24 h. *P<0.05.

To investigate the effects of glutamine metabolism on energy homeostasis in ovarian cancer cells, the cells were cultured with various concentrations of glutamine for 24 h, and cellular glucose uptake was detected with 2-NBDG fluorescence assay. The results indicated that glutamine increased glucose uptake in our ovarian cancer cell lines (Fig. 5A). We then monitored the effect of glutamine on energy flux. The levels of cellular ATP were analyzed using luminometric ATP assay after cells were treated with various concentrations of glutamine for 24 h. Our data showed that glutamine increased cellular ATP production (Fig. 5B). Given that lactate is an important product of glycolysis and the main source of energy for cancer cells, the effects of glutamine on lactate production were assessed under the same culture condition as ATP assay. The results indicated that the lactate in the culture media slightly increased upon exposure to glutamine (Fig. 5C). Compound 968, a GLS inhibitor, and 3-BP, a glycolysis inhibitor, were used to treat cells for 24 h, and cellular ATP and lactate levels in the media were assayed. ATP production markedly decreased by the inhibition of either glutaminolysis or glycolysis, and the inhibition of glycolysis reduced ATP production more profoundly in glutamine-free media than that in 2.0 mM glutamine media in all three cell lines (Fig. 5D). The inhibition of glutaminolysis or glycolysis slightly reduced lactate production in the presence or absence of glutamine (Fig. 5E). These results suggest that glucose and glutamine were major energy sources, and glutamine supported cellular ATP production in the presence of glycolysis in ovarian cancer cells.

We also analyzed the expression of key glycolytic proteins in the presence and absence of glutamine. We treated cells with different concentrations of glutamine for 24 h. The expression levels of hexokinase 1 (HK1), platelet-type phosphofructokinase (PFKP), pyruvate kinase M 1/2 (PKM1/2), and pyruvate dehydrogenase (PDH) were analyzed using Western blotting. The results showed that HK1 and PFKP either increased or demonstrated minimal changes in HEY and SKOV3 cells, whereas HK1 increased and PFKP decreased in IGROV-1 cells. PKM1/2 and PDH exhibited different changes after glutamine treatment for 24 h (Fig. 5F, G and H). These results suggest that glutamine induces alterations in glycolytic flux, and the changes in the glycolytic proteins are mainly dependent on different cell types.

### Glutamine activates the MAPK and mTOR/S6 pathways

**Figure 6:**
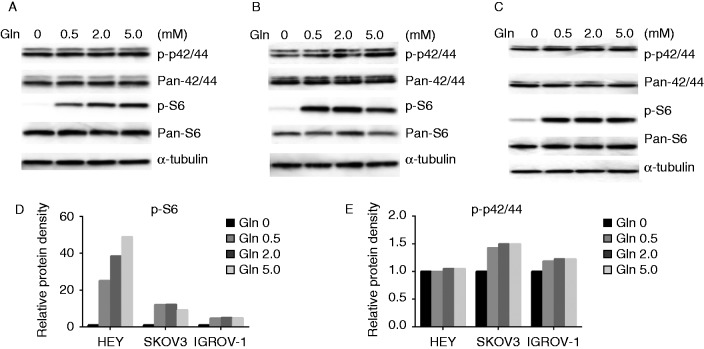
Glutamine activates the MAPK and mTOR/S6 pathways. The expression of phosphorylation of p-42/44 and p-S6 in HEY (A), SKOV3 (B), and IGROV-1 (C) cells was detected by western blotting after treatment with glutamine for 24 h. Glutamine increased protein expression of p-42/44 and p-S6 in the ovarian cancer cells (D and E).

Activation of the MAPK and phosphatidylinositol 3 kinase/Akt/mTOR pathways plays a crucial role in the control of cell growth and survival in ovarian cancer, and inhibition of these pathways leads to the inhibition of ovarian cancer growth. To investigate the mechanisms underlying the regulation of cell growth by glutamine, we characterized the effect of glutamine on the MAPK and mTOR/S6 signaling pathways. Glutamine increased phosphorylation of S6 (Ser235/236) and p42/44 (ERK) in a dose-dependent manner in ovarian cancer cells within 24 h of exposure (Fig. 6A, B, C, D and E). These results indicate that the MAPK/ERK and mTOR/S6 pathways were involved in glutamine metabolism.

### Glutamine promotes cell growth via the mTOR/S6 pathway

**Figure 7:**
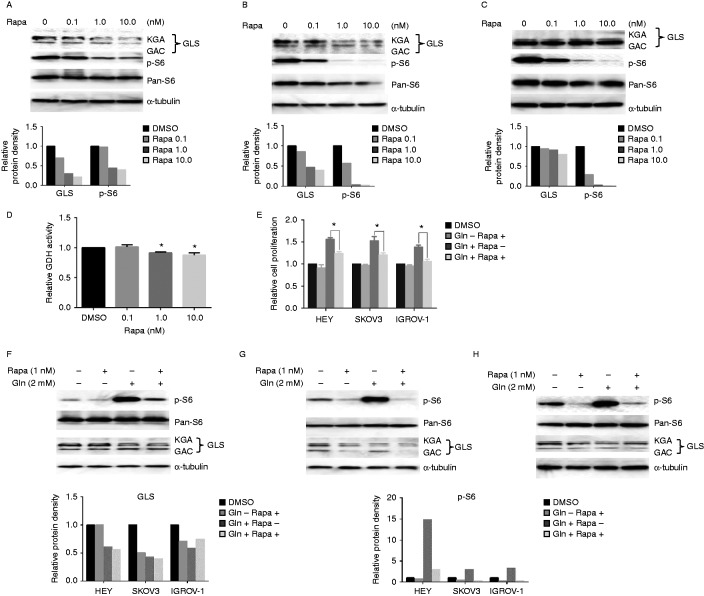
Rapamycin inhibits glutaminolysis in ovarian cancer cells. HEY (A), SKOV3 (B), and IGROV-1 (C) cells were treated with various concentrations of rapamycin as indicated in their regular media for 24 h. Treatment with rapamycin reduced the expression of GLS and phosphorylation of p-S6. Rapamycin inhibited GDH activity in HEY cells (D) and blocked the cell proliferation induced by glutamine in the ovarian cancer cells after 60 h treatment (E). Western blotting showed rapamycin inhibited the expression of phosphorylation of p-S6 and GLS induced by glutamine in HEY (F), SKOV3 (G), and IGROV-1 (H) cells after 24 h treatment. *P<0.05.

Given that glutamine strongly increased the expression of phosphorylation of S6, we investigated the relationship between the mTOR/S6 pathway and glutamine metabolism. First, we treated the three ovarian cancer cell lines with different concentrations of rapamycin (0, 0.1, 1.0, and 10 nmol/l), an mTOR inhibitor, in regular media for 24 h. The results of Western blotting showed that rapamycin strongly inhibited the expression of phosphorylation-S6 and reduced GLS expression in a dose-dependent manner (Fig. 7A, B and C). GDH activity assay also showed that rapamycin inhibited GDH activity after 24 h of treatment (Fig. 7D). We next treated cells with rapamycin in the presence or absence of glutamine conditions for 60 h to access cell proliferation. The results showed that rapamycin significantly inhibited cell proliferation and blocked the phosphorylation of S6 and expression of GLS induced by glutamine (Fig. 7E–H).

**Figure 8:**
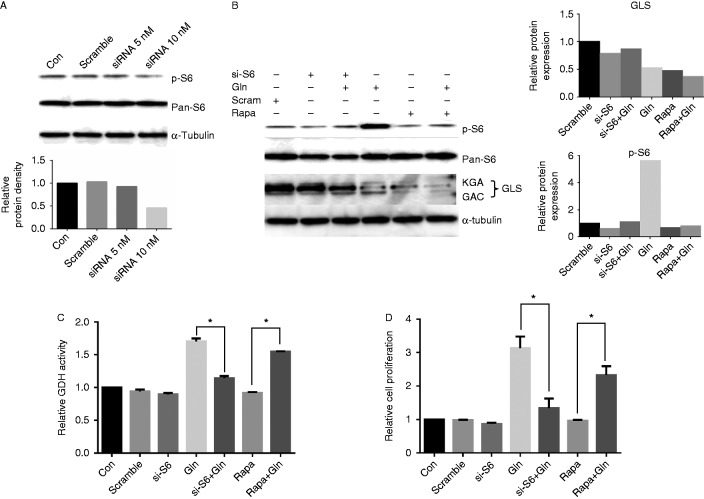
Knockdown S6 by siRNA transfection reduces the expression of GLS and activity of GDH. HEY cells were transfected with RPS6 siRNA for 24 h. Western blotting showed the expression of phosphorylation of S6 was inhibited after siRNA transfection (A). HEY cells were treated with 2.0 mM glutamine for 24 h after siRNA transfection. Both of S6 siRNA and rapamycin reduced the expression levels of GLS and phosphorylation of S6 (B). S6 siRNA transfection decreased GDH activity induced by glutamine (C). HEY cells were treated with 2.0 mM glutamine for 48 h after siRNA transfection. Cell proliferation was determined by MTT assay (D). *P<0.05.

To further confirm the role of the mTOR/S6 pathway in glutaminolysis, we transfected S6 siRNA into HEY cells to knockdown S6 protein expression. After 24 h of transfection, western blot showed that S6 siRNA effectively inhibited the phosphorylation of S6 (Fig. 8A). We then treated cells with glutamine for 24 h after siRNA transfection. The results of western blot showed that siRNA transfection inhibited the phosphorylation of S6 induced by glutamine (Fig. 8B). We further analyzed the activity of GDH, and found that siRNA transfection markedly reduced GDH activity in HEY cells treated with glutamine (Fig. 8C). Finally, we transfected S6 siRNA into HEY cells in 96-well plates, and the cells were treated with glutamine for 48 h. MTT assay showed that S6 siRNA significantly reduced cell proliferation induced by glutamine (Fig. 8D). These data suggest that glutamine metabolism is dependent on the mTOR/S6 pathway, and S6 protein is a key component involved in cell proliferation induced by glutamine in our ovarian cancer cells.

## Discussion

Even though glutamine is the most common amino acid in tissues and plasma and represents a major source of carbon and energy in cancer cells, no previous reports focus on glutamine metabolism in ovarian cancer cells from a therapeutic perspective. In this study, we investigated the potential mechanism of glutamine in modulating cell growth in human ovarian cancer cells. Our results showed that compared with glutamine deprivation, administration of glutamine improved cell proliferation through the mTOR/S6 pathway, inhibited apoptosis and cell stress subjected to depletion of glutamine, and altered glycolytic activity, which led to increased ATP and lactate production. These results are consistent with the view that targeting glutamine metabolism with molecular intervention may be used as an effective strategy for ovarian cancer therapy.

Understanding the effects of Glutamine deprivation on cell growth is necessary for full appreciation of the importance of glutamine metabolism to maintain cancer cell survival. Given that glutamine deprivation has been shown to decrease cell proliferation, DNA and protein synthesis in hepatocellular, prostate, breast and cervical cancer cells in a cell type-specific manner (Fu *et al*. 2008, Dang 2010, Ko *et al*. 2011), we chose to evaluate the possible role of glutamine deprivation on triggering of the cell cycle arrest and apoptosis. The impact of glutamine deprivation on cancer cell cycle progression and apoptosis is less well characterized. Recent *in vitro* studies have provided evidence that there are differential responses of cancer cells to glutamine deprivation under different genetic and epigenetic background (Collins *et al*. 1998, Simpson *et al*. 2012, Hensley *et al*. 2013, Phang *et al*. 2013). Cancer cells and transformed cells with c-Myc overexpression undergo apoptosis in response to glutamine limitation by intrinsic and/or extrinsic pathways depending on the cell type (Yuneva *et al*. 2007, Qing *et al*. 2012). The depletion of glutamine induced G1 phase arrest in breast and prostate cancer cells, while K-Ras-driven cancer cells and transformed cells arrested in either S- or G2/M-phase alone, with the changes triggered by glutamine deprivation (Thornthwaite & Allen 1980, Fu *et al*. 2003, Saqcena *et al*. 2013, 2015). In this study, we examined changes in the cell cycle and apoptosis in the three cell lines treated with different concentrations of glutamine for 24 h. Our results demonstrated that depletion of glutamine inhibited cell proliferation in the ovarian cancer cells via increased Annexin-V expression (Fig. 3A, B and C), and induced cell cycle G1 arrest (Fig. 2A, B and C). As a result, the expressions of cyclin D and CDK4 were down-regulated, whereas p21 was strongly enhanced (Fig. 2D, E and F), thus establishing the conditions that brought cells to a G1 cell cycle arrest. These results indicate that the anti-proliferative effects exerted by glutamine deprivation can be attributed to the induction of cell cycle arrest and apoptosis.

The active cells are constantly exposed to the natural byproducts of normal metabolism of oxygen, notably ROS, which activate signaling events that facilitate both normal and cancer cell proliferation (Weinberg *et al*. 2010). The elevated ROS productions may cause cell oxidative stress and result in significant damage to cell structures and functions. Glutamine is involved in antioxidant defense function in cells by increasing glutathione (GSH) levels, decreasing ROS levels and providing a source of NADPH, which in turn protects cells from oxidative stress (Shanware *et al*. 2011). Depletion of Glutamine has been previously found to increase the generation of ROS and reduce GSH levels in prostate cancer cells (Fu *et al*. 2006, Liu *et al*. 2011). Administration of Glutamine attenuated oxidative stress and ER stress in rats with 2,4,6-trinitrobenzene sulfonic acid induced colitis (Crespo *et al*. 2012). After treating our ovarian cancer cells with different concentrations of glutamine, we first found that glutamine resulted in decreased ROS levels induced by depletion of glutamine and was accompanied by decreased expression of ER stress markers including Calnexin, Bip, PERK, and PARP after 24 h of treatment (Fig. 4A, B, C and D). This suggests that glutamine has a function in protecting against the cell stress induced by glutamine restriction or other stress inducers. It has been reported that knockdown GLS2 (GLS) by siRNA increased ROS production and oxidative DNA damage in colon cancer cells and elevated GLS2 expression was necessary for cells to maintain intracellular levels of glutamate, α-ketoglutarate, GSH, and ROS (Hu *et al*. 2010, Suzuki *et al*. 2010). The complexity of both oxidative stress and ER stress and the mechanisms by which depletion of glutamine induced both stresses provide opportunities for further investigation.

Oxidation of glutamine's carbon backbone in the mitochondria is a major metabolic function of glutamine and a primary source of energy for proliferating cells in some cancer cells (DeBerardinis & Cheng 2010). Glycolysis and mitochondrial OXPHOS are two tightly coupled processes. The point of balance between glycolysis and OXPHOS fluctuates depending on the changes in their microenvironment and the genetic make-up of a cancer cell. Cancer cells maintain a significant level of OXPHOS capacity to rapidly switch from glycolysis to OXPHOS during carcinogenesis and cell energy stress (Antico Arciuch *et al*. 2013, Billiard *et al*. 2013). Glutamine deprivation increased glucose consumption, elevated PDH activity and decreased cellular ATP production in prostate cancer cells. Similar results have been obtained in pancreatic cancer cells and human embryonic kidney cells (HA1ER) with glutamine stimulating glucose uptake (Kaadige *et al*. 2009). Glutamine deprivation likely reduces the availability of glutamate and α-ketoglutarate in the TCA cycle, leading to a compensatory increase in the activity of aerobic glycolysis. In this study, we investigated the effect of glutamine on glycolysis in ovarian cancer cells. While decreasing both glucose uptake and cellular ATP, we discovered that depletion of glutamine increased GLS protein expression in a time dependent manner and the expressions of glycolytic proteins induced by glutamine was diverse after 24 h of treatment of glutamine in three ovarian cancer cell lines. These divergent effects may be a consequence of the different genetic backgrounds in response to different glutamine concentrations for 24 h in our cell lines. This raises the possibility that the complex metabolic coordination between glucose and glutamine metabolism observed in ovarian cancer cells is the result of genetic mutations, presumably specific to cell type (DeBerardinis & Cheng 2010).

Constitutively activated proliferative signaling pathways via genetic mutations have been recognized as a major driver for carcinogenesis in ovarian cancer (hAinmhire *et al*. 2014). The AKT/mTOR and MAPK pathways seem to be major pathways involved in the regulation of cell proliferation, invasion and bioenergetics by glutamine in some cancers including ovarian cancer (Yang *et al*. 2014). Glutamine stimulates the proliferation of pTr cells and regulates intestinal permeability and protein synthesis in intestinal epithelial cell through mTOR /S6 signal transduction pathway (Boukhettala *et al*. 2012, Kim *et al*. 2013). Additional evidence shows that glutamine flux regulates mTOR activation through facilitating the uptake of leucine (Nicklin *et al*. 2009). Studies on the regulation of cell proliferation by glutamine in the MAPK pathway are controversial (Nicklin *et al*. 2009). Previous studies demonstrated inhibition of the MEK/ERK pathway interfered with glucose metabolism but not glutamine metabolism (Traves *et al*. 2012). However, glutamine promoted growth, migration, and differentiation in HDPCs cells through activated MAPK pathway (Kim *et al*. 2014). The connection between mTOR/S6 and MAPK signal transduction pathways related to glutamine metabolism is unknown. In order to determine the effects of glutamine on the mTOR/S6 and MAPK pathways, we detected changes in the phosphorylation of ribosomal protein S6, targets of AKT/mTOR, and phosphorylation of p42/44, a key protein in MAPK pathway. Increased phosphorylated S6 and phosphorylated p42/44 protein expressions were observed in the three cell lines after treatment with glutamine for 24 h (Fig. 6A, B, C, D and E), indicating the positive effects of mTOR/S6 and MAPK signaling pathways on cell proliferation in response to glutamine in ovarian cancer cells. We further demonstrated that inhibition of mTOR by rapamycin decreased the expression of GLS protein and activity of GDH and blocked cell proliferation induced by glutamine in the three cell lines (Fig. 7A, B, C, D, E, F, G and H). Knockdown of S6 by siRNA showed that S6 is involved in the regulatory mechanism of glutamine metabolism for cell growth through GLS and GDH activities (Fig. 8B, C and D). Our results are consistent with previously published data, which confirmed that the mTOR pathway regulated glutamine metabolism by promoting the activity of GDH that required transcriptional repression of SIRT4 (Csibi *et al*. 2013).

In conclusion, the present study provides the first detailed comprehensive analysis of glutamine on cell proliferation, glycolysis, cell cycle and apoptosis, cell stress, energy flux and mTOR/S6 signaling pathways in ovarian cancer cells. Our results indicate that glutamine promotes cell growth by activating the mTOR/S6 and MAPK pathways. Therefore, based on the preliminary data generated by these studies, we believe that targeting glutamine metabolism might prove a valuable novel therapeutic strategy for ovarian cancer therapy.

## Author contribution statement

Manuscript editing: C Zhou and V L Bae-Jump. Conceived and designed the experiments: C Zhou and V L Bae-Jump. Performed the experiments: L Yuan, X Sheng, A K Willson, D R Roque, J E Stine, H Guo, C Zhou, and H M Jones. Analyzed the data: C Zhou and V L Bae-Jump. Contributed reagents/materials/analysis tools: L Yuan, X Sheng, C Zhou, and V L Bae-Jump. Wrote the paper: C Zhou and V L Bae-Jump.
